# Genome-wide association analysis reveals new targets for carotenoid biofortification in maize

**DOI:** 10.1007/s00122-015-2475-3

**Published:** 2015-02-18

**Authors:** Willy B. Suwarno, Kevin V. Pixley, Natalia Palacios-Rojas, Shawn M. Kaeppler, Raman Babu

**Affiliations:** 1 Department of Agronomy and Horticulture, Faculty of Agriculture, Bogor Agricultural University, Jl. Meranti Kampus IPB Dramaga, Bogor, 16680 Indonesia; 2 International Maize and Wheat Improvement Center (CIMMYT), Km 45 Carretera Mexico-Veracruz, Texcoco, Mexico, 56130 Mexico; 3 Department of Agronomy, University of Wisconsin-Madison, 1575 Linden Drive, Madison, WI 53705 USA

## Abstract

****Key message**:**

***Genome-wide association analysis in CIMMYT’s association panel revealed new favorable native genomic variations in/nearby important genes such as hydroxylases and CCD1 that have potential for carotenoid biofortification in maize.***

**Abstract:**

Genome-wide association studies (GWAS) have been used extensively to identify allelic variation for genes controlling important agronomic and nutritional traits in plants. Provitamin A (proVA) enhancing alleles of lycopene epsilon cyclase (*LCYE*) and β-carotene hydroxylase 1 (*CRTRB1*), previously identified through candidate-gene based GWAS, are currently used in CIMMYT’s maize breeding program. The objective of this study was to identify genes or genomic regions controlling variation for carotenoid concentrations in grain for CIMMYT’s carotenoid association mapping panel of 380 inbred maize lines, using high-density genome-wide platforms with ~476,000 SNP markers. Population structure effects were minimized by adjustments using principal components and kinship matrix with mixed models. Genome-wide linkage disequilibrium (LD) analysis indicated faster LD decay (3.9 kb; *r* ^2^ = 0.1) than commonly reported for temperate germplasm, and therefore the possibility of achieving higher mapping resolution with our mostly tropical diversity panel. GWAS for various carotenoids identified *CRTRB1,* *LCYE* and other key genes or genomic regions that govern rate-critical steps in the upstream pathway, such as *DXS1, GGPS1,* and *GGPS2* that are known to play important roles in the accumulation of precursor isoprenoids as well as downstream genes *HYD5*, *CCD1*, and *ZEP1*, which are involved in hydroxylation and carotenoid degradation. SNPs at or near all of these regions were identified and may be useful target regions for carotenoid biofortification breeding efforts in maize; for example a genomic region on chromosome 2 explained ~16 % of the phenotypic variance for β-carotene independently of *CRTRB1,* and a variant of *CCD1* that resulted in reduced β-cryptoxanthin degradation was found in lines that have previously been observed to have low proVA degradation rates.

**Electronic supplementary material:**

The online version of this article (doi:10.1007/s00122-015-2475-3) contains supplementary material, which is available to authorized users.

## Introduction

Maize is one of the three most important staple food grains worldwide and is a source of calories, protein, vitamins, and minerals in the diets of 300 million inhabitants of sub-Saharan Africa, Latin America and some parts of Asia. While the annual per capita food maize consumption in these regions averages 36, 23, and 10 kg, respectively, it exceeds 100 kg in several countries (Atlin et al. 2011). Maize-based diets tend to be deficient in the important micronutrients provitamin A (proVA), iron and zinc, and over-dependence of people on maize-based diets may result in poor health including stunted growth, reduced capacity for physical activity, and in extreme cases, high incidence of anemia, corneal blindness, compromised immunity and infant morbidity (Saltzman et al. 2013).

Most yellow maize grown and consumed throughout the world, however, has less than 2 μg g^−1^ of proVA (Pixley et al. 2013). Biofortification of maize grains with high levels of proVA carotenoids is a promising solution to overcome vitamin A malnutrition (Graham et al. 2001; Saltzman et al. 2013). ProVA maize breeding is led by the International Maize and Wheat Improvement Center (CIMMYT) and the International Institute of Tropical Agriculture (IITA) in collaboration with public- and private-sector research partners in Southern Africa and supported by the HarvestPlus Challenge Program (www.harvestplus.org). In 2012, proVA breeding efforts resulted in the release of three maize hybrids in Zambia and two in Nigeria with total proVA carotenoid concentrations of more than 7 μg g^−1^, and experimental cultivars with 10–15 μg g^−1^ have been identified (Dhliwayo et al. 2014; Saltzman et al. 2013; Suwarno et al. 2014).

The carotenoid biosynthetic pathway is well studied and the enzymes involved in carotenogenesis are documented in maize and other species (Giuliano et al. 2008; Li et al. 2009). Considerable diversity exists in the regulation of synthesis and catabolism of carotenoids (Auldridge et al. 2006; Vallabhaneni et al. 2010; Arango et al. 2014) (Fig. S1). In addition to biosynthesis and catabolism (enzymatic degradation), other factors like non-enzymatic degradation (oxidation, photo and thermal degradation), sequestration and intracellular localization of carotenoids influence their accumulation. Although knowledge of the mechanisms regulating carotenoid content and composition is increasing (Shumskaya and Wurtzel 2013; De Moura et al. 2013), it is still incomplete. Studies of carotenoid content and composition in maize grains have identified significant allelic variation for key genes such as lycopene epsilon cyclase (*LCYE*) (Harjes et al. 2008) and β-carotene hydroxylase 1 (*CRTRB1*) (Yan et al. 2010) that govern critical steps in the pathway, leading to the successful use of marker-assisted selection (MAS) in applied breeding programs (Babu et al. 2013).

Discovery efforts to understand key genes involved in natural variation for carotenoid content have used genome-wide association (GWAS) approaches to explore allelic variation at loci previously established to be associated with the carotenoid pathway in maize or other model species (Harjes et al. 2008; Vallabhaneni et al. 2009; Yan et al. 2010). With the onset of high-density genotyping platforms, like Illumina’s infinium (MaizeSNP50 at http://res.illumina.com) and genotyping by sequencing (GBS) (Elshire et al. 2011), it is now possible to quickly generate millions of marker data points that are distributed throughout the genome. GWAS based on high density, extensive marker coverage increases our ability to explain the inheritance of target traits (Gibson 2010; Stranger et al. 2011).

Our objective was to use high-density marker platforms to identify allelic variation that influences total and component carotenoids concentrations in grain for CIMMYT’s maize carotenoid association mapping (CAM) panel comprised of 380 diverse tropical, subtropical and temperate inbred lines.

## Materials and methods

### Phenotype data

The carotenoids association mapping (CAM) panel consisted of 380 diverse lowland tropical (47 %), subtropical (47 %) and temperate (3 %) lines assembled by CIMMYT’s HarvestPlus-funded maize breeding program. The panel includes 10 lines in which a proVA-enhancing allele of *CRTRB1* has been incorporated through marker-assisted selection (Babu et al. 2013). The CAM panel was grown in three environments–summer 2010 (TL10) and summer 2011 (TL11) at Tlaltizapan, Morelos, Mexico, and summer 2012 (AF12) at Agua Fria, Puebla, Mexico. Tlaltizapan is located at 18°41′ N, 99°07′ W, 945 m above sea level (masl), and has average annual temperature of 23.5 °C and average annual precipitation of 840 mm. Agua Fria is located at 20°32′ N, 97°28′ W, 110 masl, and has average annual temperature of 22 °C with average annual precipitation of 1,200 mm. Field plots were single, 5 m long rows with about 26 plants, and were unreplicated at Tlaltizapan and had two replications at Agua Fria. Two to six plants in each plot were self-pollinated and ears were collected at harvest maturity. Kernels were bulked for subsequent carotenoid analyses.

Carotenoid analyses were conducted at CIMMYT’s maize quality laboratory, Mexico. Random samples of 50 seeds were kept frozen at −80 °C until being ground to a fine powder (0.5 µm), followed by the CIMMYT laboratory protocols for carotenoids analysis, including extraction, separation, and quantification by HPLC for TL10 and TL11 environments (Galicia et al. 2008), and by UPLC for AF12 (Galicia et al. 2012). Only the separation procedure varied between the two methods, such that the HPLC method allowed better resolution for the xanthophylls (lutein and zeaxanthin) as compared to the UPLC. Lutein (LUT), zeaxanthin (ZEA), β-cryptoxanthin (βCX), β-carotene (βC), and total proVA concentrations (proVA = βC + 0.5(βCX)) were measured and reported in μg g^−1^ of kernel dry weight.

### Genotype data

Genotype data were generated through two platforms, 55 K (56,110 SNPs) and GBS v2.7 (954,179 SNPs); we used both platforms to benefit from the additional power that this might offer. The 55 K genotyping utilized the MaizeSNP50 Genotyping BeadChip from Illumina (catalog is available at www.illumina.com) and was carried out at the Syngenta facility, Slater, IA, USA, and the GBS genotyping was conducted at the Institute for Genomic Diversity, Cornell University, Ithaca, NY, USA. The physical coordinates of GBS and 55 K SNPs are derived from AGPv2. Based on the twin criterion of Call Rate (>0.85 for 55 K and >0.3 for GBS) and Minor Allele Frequency (MAF) (>0.05 for 55 K and >0.02 for GBS), we selected 39,846 SNPs from the 55 K chip and 435,975 SNPs from the GBS. We adopted different CR and MAF criterion for GBS and 55 K data owing to the nature of the genotyping platform and the ability to uncover the rare alleles. The 55 K dataset had less than 5 % missing datapoints and hence did not require imputation. On the contrary, the GBS dataset originally had close to 40 % of the datapoints missing. The GBS service provider (Institute of Genomic Diversity, Cornell University) performed a partial imputation based on an algorithm that searched for the closest neighbor in small SNP windows across the entire maize database (approximately 22,000 Zea samples), allowing for a 5 % mismatch (Romay et al. 2013). If the requirements were not met, the SNP was not imputed. A previous study reported approximately 4 % median discrepancy rates between actual and imputed calls in the Goodman association panel of maize (Romay et al. 2013). The partially imputed GBS data in our study contained 13 % missing data. The partially imputed GBS data were combined with unimputed 55 K data for further analysis. For GWAS, we combined ‘filtered 55 K’ and ‘filtered GBS’, which resulted in a combined dataset of 475,821 SNPs. From this, a subset of high quality markers (171,696 SNPs with CR >0.9 and MAF >0.1) was used for deriving PCA and kinship matrices. A large number of markers will provide greater opportunity for identifying significant associations in GWAS studies, whereas the use of the reduced marker set with less missing data and robust MAF was desirable for analysis of population structure and kinship.

### Statistical analysis

Each carotenoid trait (*y*) was transformed to log_10_(y + 1) to approach normality of residuals and equality of residual variances assumptions prior to performing analysis of variance. Distributions of phenotypic values before transformation are presented in Fig. S2. Best Linear Unbiased Estimators (BLUEs) obtained from the multi-location analyses were used in the GWAS. Pearson phenotypic correlation coefficients among carotenoid concentrations were calculated using inbred line means in log_10_(y + 1) scale, and phenotypic correlation coefficients between environments were calculated to evaluate consistency of phenotypes across the three environments. ANOVA and correlation analyses were conducted using R software (R Core Team, 2012). log_10_(y + 1) scale was used because of the presence of zero values for some of the traits. The transformed mean values were used in the GWAS analyses.

The population structure was evaluated using the K-means clustering method using the ‘adegenet’ library in R, and a principal component analysis (PCA) was performed in SNP and Variation Suite (SVS) v7.7.8 (SVS, Golden Helix, Inc., Bozeman, MT, www.goldenhelix.com). The first two PCA coordinates were used to visualize genetic distances among inbred lines, and the PCA results were then labeled using K-means groupings of the lines. An estimated number of groups in the population was obtained based on Bayesian information criterion (BIC) values (Jombart et al. 2010), visual inspection of the PCA plot and pedigree information.

The extent of genome-wide and chromosome-wise linkage disequilibrium (LD) was evaluated based on adjacent-pairwise *r* ^2^ values (the squared correlation coefficients among alleles at two adjacent SNP markers) and physical distances among these SNPs (Remington et al. 2001). Non-linear models with *r* ^2^ as responses (*y*) and pairwise distances (*x*) as predictors were fitted into the genome-wide and chromosome-wise LD data using the ‘nlin’ function in R. Average pairwise distances in which LD decayed at *r* ^2^ = 0.2 and *r* ^2^ = 0.1 were then calculated based on the model. The expected value of *r* ^2^ was:

$$E\left( {r^{2} } \right) = \left[ {\frac{10 + C}{(2 + C)(11 + C)}} \right]\left[ {1 + \frac{{(3 + C)(12 + 12C + C^{2} )}}{n(2 + C)(11 + C)}} \right]$$

where *r* ^2^ = squared correlation coefficient, *n* = sample size, and C is a model coefficient for the distance variable (Hill and Weir 1988).

Ten principal components from the PCA as obtained by SVS v7.7.8 were used as covariates in the linear models for GWAS analyses. The PCA was performed using the method implemented in the EIGENSTRAT software, in which 10 principal components are recommended as a default value for population structure correction (Price et al. 2006). A kinship matrix was generated from identity-by-state distances among inbred lines, calculated as:

IBS distance = [No. of markers IBS2 + 0.5 × (No. of markers IBS1)]/No. non-missing markers.

Where, IBS1 and IBS2 are the states in which the two inbred lines share one or two alleles, respectively, at a marker (Bishop and Williamson 1990). PCA and kinship analyses were carried out using SVS.

Individual SNP-based association tests were conducted using the correlation/trend method (Weir 2008) using SVS. Two association mapping models were used:

Y = SNP × β + PC × α + ε (Fixed effect linear model, FELM).

Y = SNP × β + PC × α + K + ε (Mixed linear model, MLM).

Where, *Y* = response of the dependent variable (means of carotenoids phenotypes across environments), SNP = SNP marker (fixed effects), PC = principal component coordinate from the PCA (fixed), *K* = kinship matrix (random), and α and β are SNP and PC fixed effect model coefficients, respectively. Traits included in the association mapping analyses were βC, βCX, ZEA, and LUT:ZEA ratio (L:Z). Another association analysis for βC was performed using the most significant SNP as a covariate. The phenotypic values (*y*) were transformed to log_10_(y + 1) scale for all traits.

Association mapping model evaluations were based on visual observation of the quantile–quantile (Q–Q) plots, which are the plots of observed −log10*P* values versus expected −log10*P* values under the null hypothesis that there is no association between marker and the phenotype. In this study, we devised two different approaches of identifying GWAS signals. The first approach is that of solely based on smallest *P* values obtained from mixed linear models, where the SNPs were ranked based on their ascending order of MLM-*P* values (smallest on top), top 40–50 SNPs were selected for each trait, grouped into 500 kb chromosome segments and then the most significant SNP in each of the top 5–10 genomic regions was identified. This approach was effective in identifying the significant associations but frequently ignored SNPs whose minor allele is not prevalent (MAF values in the range of 0.02–0.05) in the association panel but included for GWAS analyses. One of the unique and significant aspects of this study is the high-density GBS genotype data, which uncovered large number of rare alleles as compared to 55 K chip data which mostly detected common SNP alleles. Hence we adopted a second approach in which, we identified significant rare allele associations based on multiple criterion such as phenotype average for homozygous-minor-allele genotype (DD) greater (for βC, βCX, ZEA) or lower (for L:Z) than overall mean, number of DD lines ≥8 (2 % of the population), FELM and MLM *P* values <0.01. Since the phenotype means of the minor allele are typically based on smaller number of individuals, the statistical power of this approach is limited but nevertheless was useful in identifying smaller number of candidate signals for further validation in specific bi-parental populations. Fifty markers identified based on the above-mentioned criterion were grouped into 500 kb chromosome segments, and the most significant SNP in each of the top 5–10 regions was identified for each trait. It is to be noted that the physical positions of GBS-SNPs are based on B73 sequence and considering the pronounced genome non-collinearity among different maize germplasm (Xu and Messing 2006), we considered broader intervals (up to 2.5 Mb) while searching for carotenoid-related nearby genes (Table S2) in the vicinity of most significant associations.

The phenotypic difference between two homozygote classes of a given significant SNP for each of the carotenoid trait is presented as effect size, which is not corrected for population structure or kinship. To estimate the proportion of phenotypic variation explained by a combination of candidate SNPs, a multiple linear regression for each trait was performed using phenotype values as a response variable (*y*) and candidate SNPs as predictors (*x*). Best marker combinations in the model were then inferred by a stepwise selection procedure based on Akaike information criterion (AIC), using the ‘step’ function in R.

## Results

### Analyses of variance and correlations

There was significant variation among inbred lines for all carotenoids (*P* < 0.01) (data not shown). The broad-sense heritability estimates were high and ranged from 0.89 to 0.93 in the multi-location analyses for various carotenoid traits (LUT—0.89, ZEA—0.93, L:Z—0.92, Βcx—0.91, βC—0.92 and ProVA—0.93). Pearson correlation coefficients among carotenoid concentrations were mostly significant (*P* < 0.01), except for LUT with βCX, βC, and proVA (Table S3). Strong correlations (*r* ≥ 0.60) were observed between βCX and ZEA, and for proVA with βCX and βC. Correlation coefficients among environments for each trait were significantly large (*r* > 0.75, *P* < 0.01) except for AF12 (UPLC) with TL10 and TL11 (HPLC) for LUT, indicating that the carotenoid phenotypes were generally consistent across environments (Table S4). We used HPLC data for LUT and the combined (HPLC and UPLC) data for all other component carotenoids in the association analyses. Though UPLC and HPLC values largely agreed with each other in general, we observed some discrepancy particularly for LUT. One of the possible reasons for this is that the extraction and separation protocols are optimized for higher recovery of provitamin A carotenoids (βCX and βC) as compared to LUT and ZEA.

### Population structure and linkage disequilibrium

**Fig. 1:**
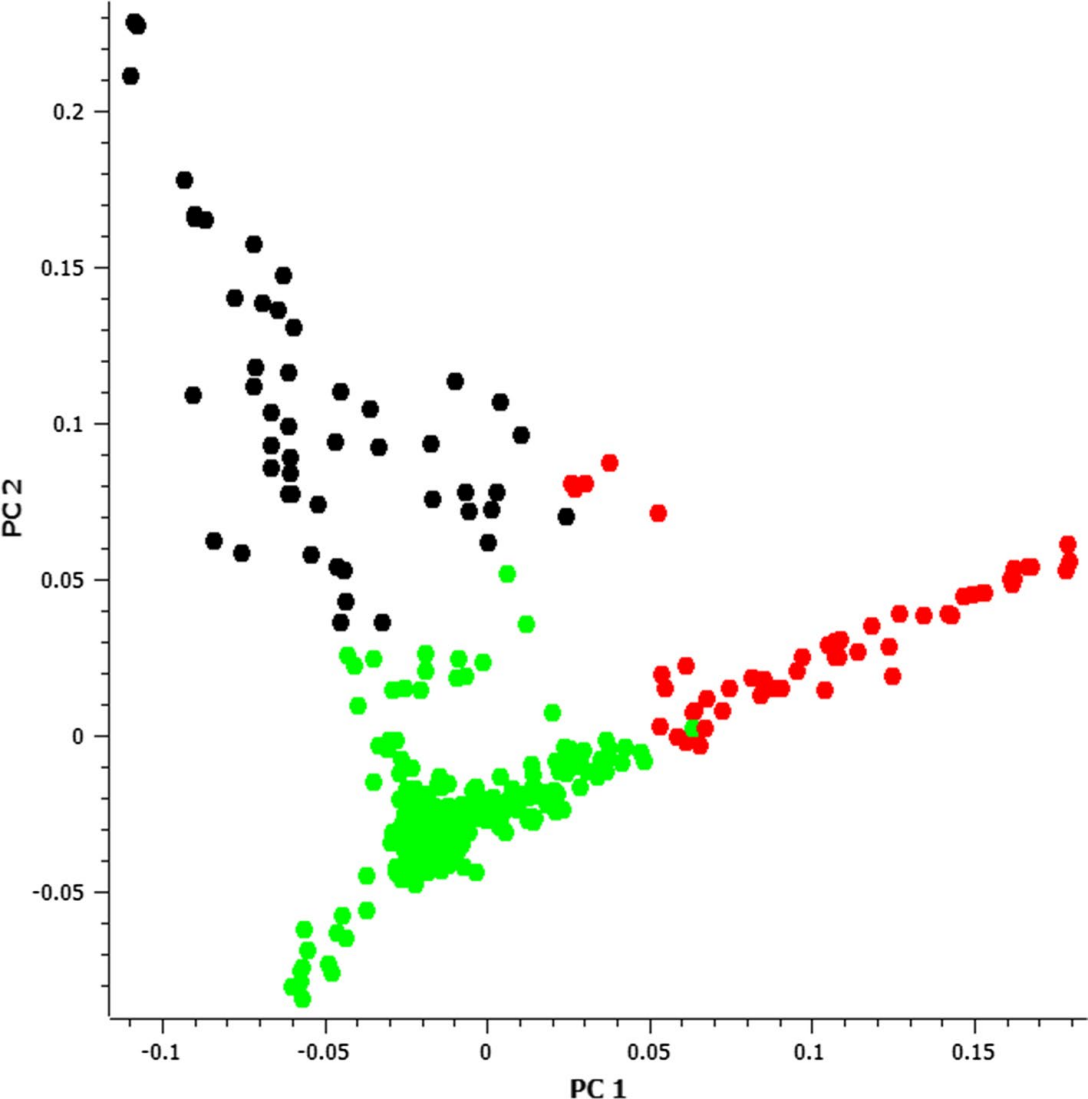
Principal coordinate analysis plot based on the GBS + 55 K data, color-labeled based on the K-means clustering results. *Black, red, and green color* represent group 1 (tropical heterotic group B lines), 2 (provitamin A source lines), and 3 (tropical heterotic group A lines), respectively

The population structure among the 380 lines was well described by the K-means clustering method (Fig. 1), where BIC model selection and visual observation of the PCA plot indicated that three clusters were most likely for this population. Group 1 (Fig. 1, colored in black) consisted of 49 lines that predominantly belonged to CIMMYT’s tropical germplasm of heterotic group B, group 3 (green) contained 277 lines from CIMMYT’s tropical germplasm of heterotic group A, and group 2 (red) included 54 lines which were mostly source germplasm for enhanced proVA content and included introgression of tropical or temperate germplasm that is exotic to CIMMYT’s breeding program. Proportions of phenotypic variation explained by this population structure alone were 24 % for βC, 17 % for βCX, 8 % for ZEA, and 10 % for L:Z.

Average physical distance between pairs of markers was 14.1 kb and the average genome-wide LD (*r* ^2^) obtained based on adjacent pairs of markers was 0.41. Genome-wide LD decay was 1.36 kb at *r* ^2^ = 0.2 and 3.89 kb at *r* ^2^ = 0.1 (Fig. S4). Chromosome-wise LD analyses showed that the slowest LD decay was observed on chromosome 8 (8.19 kb, *r* ^2^ = 0.1), followed by chromosome 4 (5.20 kb, *r* ^2^ = 0.1), whereas the remaining chromosomes had very similar average distance of 3.55 kb at *r* ^2^ = 0.1

Correcting for population structure using either PCA or the kinship matrix was important to improve predictions relative to the model involving only genotypes (the G model). Using both PCA and the kinship matrix in the model (G+Q+K or MLM model) improved the accuracy further by eliminating more false positives in the association mapping results (Fig. S6).

### Association mapping

**Fig. 2:**
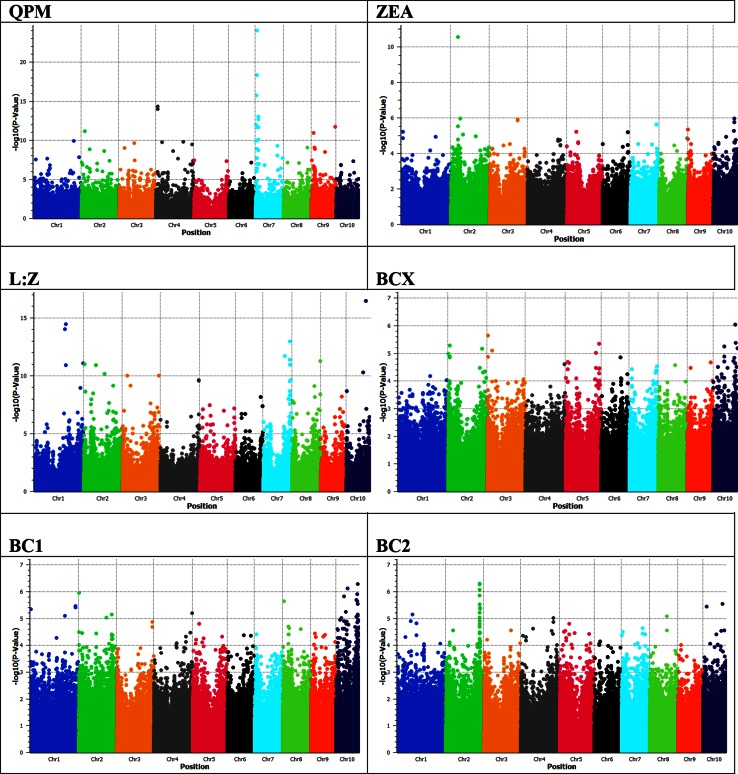
GWAS manhattan plots using the mixed linear (G+Q+K) model and the 55 K + GBS combined genotype data. *QPM* quality protein maize (binary phenotype), *ZEA* zeaxanthin, *L:Z* lutein:zeaxanthin ratio, *BCX* β-cryptoxanthin, B*C1* β-carotene, *BC2* β-carotene with the S10_135911532 marker as an additional covariate in the model. All carotenoids’ phenotypic values (*y*) were transformed to log_10_(y + 1) prior to analyses

The 55 K dataset had smaller proportion of SNPs with low minor allele frequency (MAF <0.2 = 38 %) than the GBS (58 %) and the 55 K+GBS (56 %) datasets (Fig. S3). In the latter set, which was used for association mapping, marker-free intervals ranged from 1 bp to 955.78 kb with a median of 30 bp, and SNP density was 1 SNP per 4.39 kb. The CAM panel of 380 lines had an average heterozygosity rate of 0.06, reflecting that most of these lines were either fixed or in advanced inbreeding generations. The panel included 12 QPM (quality protein maize) lines that have enhanced protein quality due to the presence of the homozygous recessive allele of the *opaque2* gene (Atlin et al. 2011). Association analysis using the QPM trait as binary phenotype (0 for normal and 1 for QPM) rightly identified the *opaque2* gene on chromosome 7, thereby validating our methods and statistical approach (Fig. 2).

Based on the mixed linear model (MLM) association analysis, significant genomic regions for each of the carotenoid component traits were identified based on smallest *P* values as well as rare alleles of large effect sizes as described in materials and methods (Table 1).

**Table 1:** The list of significant genomic regions identified through GWAS analyses for each of the component carotenoid trait

Trait/marker	Chr	Position (Mb)^a^	Minor allele	MAF	FELM *p* value	MLM *p* value	Phenotype effect (DD-dd)	*R* ^2^	No. of SNPs^b^	Nearby carotenoid gene^c^
βC										
S10_135911532	10	135.9	A	0.34	1.73E-04	5.05E-07	2.11	0.07	6	*CRTRB1*
S8_8887117	8	8.9	G	0.07	2.20E-06	2.23E-06	−1.07	0.06	1	
S10_71741924	10	71.7	T	0.03	6.71E-08	7.54E-07	10.94	0.06	3	
S10_133820657	10	133.8	C	0.02	6.70E-09	1.23E-06	11.65	0.06	1	*CRTRB1*
S10_134655704	10	134.7	C	0.03	5.13E-09	2.20E-06	11.64	0.06	2	*CRTRB1*
S1_280088079	1	280.1	G	0.03	3.87E-06	3.40E-06	2.26	0.06	6	
S2_212648728	2	212.6	A	0.04	–	4.86E-07	2.98	0.16	20	
βCX										
S10_137426599	10	137.4	A	0.09	1.58E-05	4.11E-06	−1.68	0.06	1	*CRTRB1*
S5_203698331	5	203.7	T	0.32	4.95E-05	4.49E-06	−1.36	0.06	1	
S2_9365226	2	9.4	G	0.14	2.86E-07	5.08E-06	−1.28	0.06	3	
S10_67113044	10	67.1	A	0.35	2.24E-07	5.58E-06	−1.18	0.05	3	
S10_146965726	10	147.0	C	0.05	7.86E-05	6.48E-06	−2.03	0.05	1	
S2_207044142	2	207.0	C	0.12	6.62E-06	6.68E-06	1.46	0.05	1	*GGPS1*
S3_30009999	3	30.0	G	0.30	1.23E-05	7.83E-06	−1.08	0.05	1	
S5_184160168	5	184.2	T	0.30	1.13E-04	9.32E-06	0.66	0.05	4	
S2_16916669	2	16.9	C	0.14	4.86E-03	1.04E-03	2.45	0.03	1	*HYD1*
S9_151998412	9	152.0	A	0.05	5.39E-04	2.95E-03	2.47	0.02	1	*CCD1/HYD5*
S10_133820657	10	133.8	C	0.02	2.86E-08	9.14E-07	−2.27	0.06	1	*CRTRB1*
S3_5777896	3	5.8	C	0.04	1.86E-04	2.23E-06	−1.68	0.06	2	
S6_146033807	6	146.0	G	0.03	7.27E-04	1.23E-03	3.14	0.03	3	*DXS1*
ZEA										
S2_44448492	2	44.4	G	0.31	1.85E-14	2.77E-11	−5.12	0.11	3	*ZEP1*
S2_57643868	2	57.6	G	0.06	3.07E-07	1.04E-06	−4.98	0.06	1	
S3_172381448	3	172.4	C	0.07	4.78E-08	1.20E-06	−5.22	0.06	3	
S9_566438	9	0.6	G	0.05	5.36E-06	4.38E-06	−5.19	0.06	3	
S10_125724462	10	125.7	C	0.05	4.07E-07	5.29E-06	−5.31	0.05	2	
S1_8945297	1	8.9	T	0.47	3.44E-06	5.76E-06	−1.94	0.05	3	
S6_154891169	6	154.9	C	0.11	9.95E-06	6.24E-06	−4.09	0.05	1	
S9_133887810	9	133.9	G	0.05	2.22E-04	3.75E-03	9.41	0.02	4	
S9_151998412	9	152.0	A	0.05	3.5E-04	9.95E-03	2.86	0.02	1	*CCD1/HYD5*
S10_126915113	10	126.9	G	0.04	6.19E-09	1.06E-06	−6.03	0.06	3	
S7_160069429	7	160.1	A	0.03	1.03E-06	2.24E-06	−5.63	0.06	1	*GGPS2*
S5_58706973	5	58.7	G	0.03	1.49E-04	5.91E-06	−5.27	0.05	1	
S5_196023723	5	196.0	G	0.03	3.45E-05	4.31E-04	10.37	0.03	11	
S7_124945321	7	124.9	A	0.03	4.45E-04	9.29E-04	11.44	0.03	1	
L:Z										
S1_175591561	1	175.6	A	0.49	4.37E-05	9.78E-03	−0.41	0.02	2	
S9_130410559	9	130.4	C	0.13	2.04E-04	1.81E-03	−0.38	0.03	2	
S7_143524503	7	143.5	C	0.18	3.08E-03	6.69E-03	−0.36	0.02	9	
S3_141214784	3	141.2	T	0.34	2.11E-04	8.72E-04	−0.37	0.04	2	
S1_296844851	1	296.8	C	0.04	9.85E-08	7.97E-12	5.26	0.15	5	
S8_138523563	8	138.5	A	0.03	4.25E-14	7.25E-10	2.43	0.12	1	*LCYE*

*βC* β-carotene, *βCX* β-cryptoxanthin, *ZEA* zeaxanthin, *L:Z* lutein:zeaxanthin ratio, *Chr* chromosome, *MAF* minor allele frequency, *FELM* fixed effect linear model, *MLM* mixed linear model, *DD* homozygous-minor-allele genotype, *dd* homozygous-major-allele genotype

^a^The exact physical position of the SNP can be inferred from marker’s name, for example, S10_135911532: chromosome 10; 135,911,532 bp

^b^Number of significant SNPs within a 500 kb window

^c^The nearest previously identified carotenoid-pathway-related gene

Among the seven significant associations for β-carotene (βC), four were located on chromosome 10, and the most significant one (S10_135911532) was located near (vicinity of 500 kb) the previously identified *CRTRB1* gene. Two other SNPs with significant *P* values on chromosome 10 (S10_133820657 and S10_134655704) were also located in the vicinity of *CRTRB1,* with large effect sizes (~11 µg g^−1^) and small MAF (0.02–0.03), which was in line with our expectation as there were only 10 *CRTRB1* introgression lines in the panel. Interestingly, we found a genomic region on chromosome 2 (S2_212648728) associated with βC concentrations after accounting for the variation explained by the most significant marker (S10_135911532/*CRTRB1*). This region was highlighted conspicuously by multiple significant SNPs in the Manhattan plot (Fig. 2), in which the most significant SNP in the region had an effect of ~3 µg g^−1^ and explained close to 16 % of phenotypic variance for βC. Rare allele analysis was conducted to identify genomic regions where minor alleles had favorable effects; however, rare allele analysis for βC did not reveal any additional regions that were unrelated to *CRTRB1*. A linear multiple regression model with five most significant SNPs explained approximately 61 % of the phenotypic variance for βC (*P* < 0.01; Table 2).

**Table 2:** Multiple linear regressions of carotenoids (*y*) using selected SNPs (*x*) based on MLM *p* values and rare allele analysis

Trait	Candidate SNP analysis^a^	No. of SNPs in the model^b^	No. of lines	F	*P* value	Adjusted *R* ^2^
βC	MLM	5	122	39.58	<0.01	0.61
βCX	MLM	4	176	19.46	<0.01	0.30
	Rare allele	3	112	8.82	<0.01	0.17
ZEA	MLM	6	109	22.03	<0.01	0.54
	Rare allele	3	268	11.55	<0.01	0.14
L:Z	MLM	1	121	65.24	<0.01	0.35
	Rare allele	3	150	6.65	<0.01	0.22

*βC* β-carotene, *βCX* β-cryptoxanthin, *ZEA* zeaxanthin, *L:Z* lutein:zeaxanthin ratio, *MLM* G+Q+K model

^a^Analysis from which 10 candidate SNPs selected

^b^Number of SNPs in the final models resulted from further selection using stepwise approach based on AIC values

The most significant marker (S10_133820657) for β-cryptoxanthin (βCX) was located in close proximity with *CRTRB1* and its minor allele frequency (0.02) matched with the number of *CRTRB1* introgression lines in the panel. The negative effect (−2.27 µg g^−1^) of this SNP was due to the negative correlation between βC and βCX. A total of 13 genomic regions with favorable minor allele were identified, of which three were rare allele type (MAF <0.05). The effect sizes of significant SNPs ranged from 0.7 to 3 µg g^−1^, each explaining 2–6 % of phenotypic variance for βCX (Tables 1 and 2). Four selected SNPs from *P* value based association analysis explained close to 30 % of phenotypic variance for βCX concentration (Table 2).

A region represented by S2_16916669, located relatively close to the non-heme di-iron β-carotene hydroxylase gene *HYD1* (2: 15,865,938–15,868,219), and another by S9_151998412, located near both the carotenoid cleavage dioxigenase 1 *CCD1* (9: 152,086,899–152,092,882) and *HYD5* (9: 153,692,212–153,694,576), suggested possible roles for these candidate genes in influencing βCX concentrations. A SNP, S6_146033807 uncovered by the rare allele analysis was located close to DXS1 gene which is involved in carotenoid metabolism.

The most significant marker (S2_44448492) for ZEA was located within the zeaxanthin epoxidase 1 (*ZEP1*) gene on chromosome 2 (44,440,299–44,449,237). The SNP had MAF of 0.31, phenotypic effect of 5.12 μg g^−1^, and explained 11 % of the phenotypic variance for ZEA concentration. Seven other candidate SNPs from *P* value based analysis had negative effects as well, indicating the prevalence of favorable alleles at most of the detected loci for ZEA in the CAM panel. Rare allele analysis revealed several candidate regions (on chromosomes 5, 7, and 10) associated with ZEA concentration. These SNPs had relatively large effect sizes, ranging from 5.2 to 11.4 µg g^−1^, and each explained 3–6 % ZEA variation. A SNP on chromosome 7 (S7_160069429) was located close to GGPS2, which is an upstream gene involved in carotenoid metabolism. Two regions on chromosomes 5 and 7 (S5_196023723 and S7_124945321) identified in the rare allele analyses were distinctly different because of their large and positive contribution of minor allele towards ZEA concentration. A linear model with six candidate SNPs from *P* value and rare allele based analyses explained close to 54 % of phenotypic variance for ZEA (Table 2).

The seven significant regions for lutein:zeaxanthin ratio (L:Z) were distributed on chromosomes 1, 3, 7, 8, and 9 (Table 1). A SNP on chromosome 8 (S8_138523563) was located within 500 kb of the *LCYE* gene and explained 12 % of phenotypic variance for L:Z. In five of the seven genomic regions, minor allele had favorable effect of lowering the L:Z ratio. Low minor allele frequency with large negative effects (difference between phenotypic averages of minor and major allele) for L:Z ratio are desired for proVA breeding because they represent opportunities to increase flux to the β branch of the carotenoid pathway (Fig. S1).

## Discussion

Genotype by environment interaction (GEI) effects can influence QTL and association mapping results, requiring the effect of identified genomic regions to be estimated for each environment (Zhang et al. 2008; Tétard-Jones et al. 2012). In the current investigation, the correlation coefficients among environments were highly significant (Table S3), indicating a minor role of GEI towards expression of most of the carotenoid component traits, as previously shown by Suwarno et al. (2014).

The phenotypic correlation coefficients among different component carotenoids were generally as expected based on their known relationships in the carotenoid biosynthetic pathway (Farré et al. 2010). Lutein, which is on the α-branch, was not significantly associated with βC or βCX which are on the β branch (Fig. 1). The significant correlation between LUT and ZEA (*r* = 0.38, *P* < 0.01) suggests that these traits increase or decrease in parallel for genotypes with more or less total flux through the carotenoid pathway. There was a strong relationship between βCX and ZEA (*r* = 0.65, *P* < 0.01), indicating significant consistency in the rate of bioconversion of the former to the latter, which is located downstream in the β-branch of the pathway (Fig. S1).

The extent of LD in any given association panel has profound influence on GWAS results because larger LD blocks and slower rate of LD decay generally result in lower mapping resolution. We found rapid LD decay (~1.4 kb, *r* ^2^ = 0.2) in CIMMYT’s predominantly tropical and subtropical CAM panel using GBS data, which however varied among different chromosomes (Figure S5). A recent study using GBS data for 2,815 inbred lines from worldwide maize breeding programs found LD of 1 kb, for tropical germplasm and 10 kb, for temperate maize (Romay et al. 2013). Lu et al. (2011) estimated considerably larger LD of 5–10 kb, and 10–100 kb for tropical and temperate germplasm, respectively, using Illumina’s golden gate genotyping platform, which used a much smaller number (1,943 SNPs) of markers relative to GBS. These studies indicated that LD decayed faster in tropical than in temperate germplasm, suggesting a wider genetic base resulting from more generations of divergence in tropical germplasm. As a consequence, higher mapping resolution using tropical germplasm is expected. Fewer markers should result in more unrepresented genomic regions, whereas more SNPs from the GBS platform should aid in the more precise estimation of LD decay distance and finer delimitation of genomic intervals for the carotenoid traits.

Most of the significant association signals for βC in our study pointed to *CRTRB1* on chromosome 10, which is known to play a significant role in enhancing βC content in a range of temperate and tropical germplasm (Yan et al. 2010; Babu et al. 2013). This result may have been driven by the inclusion of 10 S2 or S3 lines in the CAM panel which are *CRTRB1* introgressions and have average βC concentrations significantly larger than the population mean. However, the finding that the delimited genomic region was very large may have been due to smaller number of recombinations and consequently larger *CRTRB1* introgression blocks in these early generation lines. A localized LD analysis in the CRTRB1 genomic region (133–136.5 Mb) confirmed the above inference, which revealed higher LD levels (*R* ^2^ = 0.86, *D*’ = 0.97) as compared to the genome-wide average (*R* ^2^ = 0.41, *D*’ = 0.81). By comparison, GWAS analysis for the binary QPM trait using the panel’s 19 quality protein maize (QPM) inbred lines, which were derived through multiple rounds of selfing, identified the *opaque2* gene (chromosome 7: 10.79 Mb) with a resolution of ~200 Kb.

By controlling the variation explained by *CRTRB1*, we identified a significant region on chromosome 2 (S2_212648728, MAF = 0.04) that explained 16 % of phenotypic variance for βC and the the minor allele had a favorable effect of ~3 µg g^−1^. This SNP is located within the Amelogenin gene (chromosome 2: 212,648,384–212,649,098) and nearby the 40S ribosomal protein S9 gene (chromosome 2: 212,644,796–212,647,696). Seven of eight donor lines identified for this region were different from those for *CRTRB1* and belonged to diverse backgrounds including tropical, subtropical and temperate germplasm. The 40S ribosomal protein S9 gene has larger expression (FPKM = 2104.3) than that of *CRTRB1* (FPKM = 1148.5) in the endosperm at 16 days after pollination (DAP) (Sekhon et al. 2013). These results suggest potential value as an additional target region for enhanced βC content in proVA breeding programs subject to validation in bi-parental or other independent association populations.

For βCX, two of the 13 associations identified surrounded the *CRTRB1* region on chromosome 10 and the minor allele at these loci reduced the βCX content by 1.7–2.3 µg g^−1^. *CRTRB1* specifically controls hydroxylation of βC to βCX in maize endosperm tissues, and its alleles with reduced hydroxylation activity are associated with increased βC and decreased βCX content (Vallabhaneni et al. 2009; Yan et al. 2010; Babu et al. 2013). The minor alleles at eight of the detected associations decreased the βCX content, whereas they had an enhancing effect at the rest of the five regions. Interestingly, four of the five regions, in which minor alleles had a favorable trait enhancing effect overlapped with five candidate genes viz., GGPS1, HYD1, CCD1, HYD5 and DXS1, all of which have been previously demonstrated to be associated with carotenoid metabolism.

The maize genome has two types of carotene hydroxylases – one each of the P450 heme-thiolate CYP97A and CYP97C, and six unlinked paralogs of non-heme di-iron carotene hydroxylases (HYD) (Vallabhaneni et al. 2009). *HYD5* encodes an enzyme with hydroxylase domains and plastid-targeting signals, and its role has been suggested to be in the conversion of βCX to ZEA (Sun et al. 1996). If alleles of *HYD5* indeed reduce the hydroxylation of βCX to ZEA, they may present opportunities to enhance βCX and proVA concentration. Dhliwayo et al. (2014) discuss evidence that βCX has greater nutritional value, including proVA value, than commonly reported and therefore, selecting for βC-enhancing alleles of *CRTRB1* may be less desirable than a strategy of selecting for alleles that favor increased accumulation of βCX. Much research is needed to understand the specific roles of the carotene hydroxylase paralogs in the regulation of carotenoid biosynthesis and whether they may offer opportunities for breeding enhanced proVA concentrations.

By functional associations of QTL detected in two maize populations, Kandianis et al. (2013) recently concluded that total carotenoid concentration is influenced by the allocation of carbon substrates to the carotenoid pathway and by the removal of carotenoids through *CCD1*-facilitated catabolism or *ZEP*-mediated conversion. Joint linkage analysis for visually scored kernel color intensity in ten NAM (nested association mapping) populations also revealed QTL on chromosomes 2 and 9 that coincided with *ZEP1* and *CCD1* (Chandler et al. 2013). Vallabhaneni and Wurtzel (2009) previously established that the *ZEP* genes affect the conversion of ZEA to violaxanthin (a precursor of abscisic acid in maize endosperm) and negatively correlate with total carotenoid accumulation in maize endosperm. The most significant SNP for ZEA in our study resided inside the *ZEP1* gene on chromosome 2 (Table 1); replacing the unfavorable minor allele of *ZEP1* with the favorable one, therefore, may benefit many lines. A recent GWAS study in an inbred association panel of 281 lines ranging from light yellow to deep orange identified genomic variations within the coding region of *ZEP1* as one of the important determinants of seed carotenoid content, besides LUT1, LUT5 and DXS2 (Owens et al. 2014).

Two other genes that have been implicated in carotenoid metabolism (Wurtzel et al. 2012), *GGPS2* (on chromosome 7) and *DXS1* (on chromosome 6), are located in the vicinity of the 10 most significant associations that we found for ZEA (*GGPS2*) and βCX (*DXS1*). Deoxyxylulose synthase (*DXS*), together with deoxyxylulose reductase convert the three-carbon molecules from glycolysis to methyl-erythrol phosphate (MEP) (Fig. S1). The MEP pathway synthesizes isoprenoids through different enzymatic reactions including GGPS, producing GGPP, the isoprenoid substrate for carotenoid biosynthesis (Fig. S1). Notably, in the current study, the minor allele for GGPS2 was the unfavorable one (decreasing the ZEA content by ~6 µg g^−1^), indicating the prevalence of favorable alleles in the CAM panel. On the other hand, the minor allele for DXS1 was the favorable one (increasing the βCX content by ~3 µg g^−1^), and there are eight lines in the panel having homozygous favorable allele for this gene (Table S5).

The mechanisms regulating carotenoid content, including localization of carotenoid biosynthetic enzymes in amyloplasts, carotenoid catabolism and degradation are not well understood (Shumskaya and Wurtzel 2013; De Moura et al. 2013). Catabolism of carotenoids plays an important role, at least in photosynthetic tissues, to help maintain carotenoids at physiologically important levels. Although non-enzymatic degradation of carotenoids also occurs, catabolism mediated by the carotenoid cleavage enzymes (*CCD1, CCD4, CCD7* and *CCD8*) and the 9-cis-epoxycarotenoid dioxygenases (*NCED2, NCED3, NCED5, NCED6* and *NCED9*) in seeds affect carotenoid composition and content (Gonzalez-Jorge et al. 2013). CCD family genes have been shown to deplete the carotenoid pool in Arabidopsis seeds, chrysanthemum flowers and strawberries (Auldridge et al. 2006; Ohmiya et al. 2006; Vogel et al. 2008; García-Limones et al. 2008; Gonzalez-Jorge et al. 2013). In maize endosperm, correlation of *CCD1* transcript abundance with lower levels of carotenoids and a pronounced dosage effect resulting from copy number variation has been reported (Vallabhaneni et al. 2010). In addition, genotypic variation on the transcript profiles not only of *CCD1* but also of other carotenoid catabolic enzymes, *CCD4, CCD7, NCED1* and *NCED9* was observed in maize endosperm (Vallabhaneni et al. 2010). Genotypic variation has also been observed for the degradation rate of proVA carotenoids in maize; 40–70 % proVA loss has been recorded among 10 lines and hybrids after storage for 4 months at tropical ambient conditions (N. Palacios-Rojas, unpublished data).

14 significant genomic regions were identified for ZEA concentration, of which five were rare allele type associations. The confidence interval of one of the associations on chromosome 9 (S9_151998412) contained *CCD1* [linked to *WC1* locus (White Cap 1)]. We identified 11 lines that possess a native variation linked to *CCD1* on chromosome 9 (Table S6). Interestingly, one of these 11 lines is very closely related to other lines previously identified as “low degradation lines” based on observations of proVA loss during storage. In addition, experimental hybrids with some of the lines identified here with the native variation for *CCD1* in their pedigrees have also shown reduced loss of proVA (10–15 %) during one month storage compared to other hybrids that lost up to 36 % of proVA (N. Palacios-Rojas, unpublished data). Other than CCD1-related association, three regions on chromosomes 5, 7 and 9 appeared interesting, whose minor alleles had large positive effects on ZEA concentration ranging from 9.4–11.4 µg g^−1^.

Gonzalez-Jorge et al. (2013), using linkage mapping and GWAS for seed carotenoid content identified that the plastid-localized *CCD4* is a major determinant while *CCD1* has a limited contribution to dry seed βC retention in *Arabidopsis*. If such a relationship holds true for maize it could help explain the association between βCX and *CCD1* in our study (Table 2) if, for example, *CCD1* cleaves ZEA preferentially to βC and βCX. Upon validation of the favorable alleles of *CCD1* and other three candidate associations for enhanced ZEA concentration in appropriate bi-parental populations, they could be useful in proVA biofortification breeding programs as donor lines for minimizing carotenoid catabolism as well as enhancing the total flux into the β- branch of the pathway.

One of the significant markers for L:Z ratio (S8_138523563) was very closely linked to *LCYE*, which has a significant effect on relative carotenoid concentrations in the α- versus β-branches of the carotenoid pathway (Harjes et al. 2008). Other than this, four genomic regions were identified wherein the minor allele had a favorable effect of reduced L:Z ratio. The effect of these minor alleles and consequent value for selection in breeding programs requires validation in appropriate bi-parental populations. Altering the L:Z ratio, however, will not necessarily achieve the desired effect on total proVA concentration as evidenced by reports that both the α- and β-branches can be “over loaded,” resulting in feedback inhibition to total flux into the carotenoid pathway (Babu et al. 2013; Arango et al. 2014).

Besides *P* value based most significant associations for each of the carotenoid traits, we have identified rare alleles (MAF <0.05 but >0.02) that have large and favorable phenotypic effects in this study. These candidate associations are detected with less statistical power (as compared to top most *P* value based associations) but nevertheless help in identification of smaller set of candidates that could be validated in subsequent studies using bi-parental populations. GWAS based on common variants tend to ignore such rare alleles, which are now being speculated to play a crucial role in addressing the missing heritability issue (Stranger et al. 2011). We propose a four-step sequential strategy for identification and verification of such rare allele associations—(1) identify the rare variant associations at certain threshold significance (we adopted MLM-P <0.01 as nominal), (2) filter based on effect size (30 % more favorable than the population average for the trait), (3) prioritize large-effect rare variants based on evidences such as underlying candidate genes that were previously shown to be associated with the trait in either maize or other model species, and (4) identify or create suitable bi-parental populations that segregate for candidate rare variant associations and validate the phenotypic effect. Such an approach is especially relevant when the frequency of desired trait occurrence, for instance, enhanced carotenoid concentrations in our study, in the association panel is less. In the carotenoid panel that we examined, average proVA concentration was around 4 µg g^−1^ and only 16 out of the ~380 lines (less than 5 %) possessed proVA concentrations of above 8 µg g^−1^.

A genome-wide atlas documenting different spatial and temporal patterns of transcription of genes has been developed for maize using microarrays and RNA sequencing (Sekhon et al. 2011; Hansey et al. 2012; Sekhon et al. 2013). Comparison of the significant associations discovered in the current study with endosperm expression values of genes at 16 DAP (Sekhon et al. 2013) revealed at least 24 genes that were expressed, of which four (*CRTRB1, GGPS1, GGPS2,* and *CCD1*) are involved in carotenoid metabolism (Table S7). One or more of the remaining 20 genes might be important through epistatic effects on carotenoid pathway genes, for example the 40S ribosomal protein S9 gene.

The ability of multiple linear regression models to explain large proportions of the phenotypic variances (Table 2) suggests that simple assays involving a small number of SNPs could be designed for selecting lines with favorable alleles for carotenoids concentrations.

## Conclusions

GWAS suggested several genes that are not within the carotenoid pathway but may affect carotenoids concentrations, validated the significance of *CRTRB1*, and identified hydroxylase genes (such as *HYD5* and *HYD1*) and favorable native genomic variations that may help achieve higher amounts of nutritionally useful carotenoids. Detection of significant association signals in the current investigation pertaining to other candidate genes such as *GGPS2*, *DXS1, ZEP1,* and *CCD1* support the possibility of increasing proVA in maize by selecting for alleles that enhance the total biochemical flux towards carotenoid biosynthesis or arrest carotenoid catabolism in the pathway. Unfortunately, although perhaps promising for other proVA breeding programs, opportunities to enhance total flux by selecting favorable alleles of *GGPS2* and *ZEP1* appear limited in the germplasm represented by CIMMYT’s CAM panel, where the favorable alleles are already in high frequency. Focus on reduced carotenoid catabolism by selecting favorable alleles of *CCD1*, however, could contribute to proVA biofortification strategies and native genomic variations at or close to these loci have been characterized and corresponding donor lines identified for possible use in maize breeding programs aimed at carotenoid biofortification. Future research is also warranted to validate the effects and explore the utility of selecting for genes whose increased expression rates (reported by Sekhon et al. 2013) were associated with minor alleles that favorably affected carotenoids concentrations in CIMMYT’s CAM panel.

### **Author contribution statement**

RB, KP and NP—conceived the experiment; NP and KP—conducted the field evaluations and carotenoid phenotyping; RB—coordinated the 55 K and GBS experiments; WBS and RB—carried out the GWAS analyses; RB, WBS, KP, NP and SMK—interpreted the results and drafted the manuscript.

## Electronic supplementary material

Below is the link to the electronic supplementary material.

Supplementary material 1 (XLSX 23 kb)

Supplementary material 2 (DOCX 243 kb)

## Abbreviations

LUT: Lutein
ZEA: Zeaxanthin
L:Z: Lutein:zeaxanthin ratio
βCX: β-cryptoxanthin
βC: β-carotene
proVA: Total provitamin A
GBS: Genotyping-by-sequencing
LD: Linkage disequilibrium
FELM: Fixed effect linear model
MLM: Mixed linear model
MAF: Minor allele frequency
MEP: Methyl-erythriol 4-phosphate
DMAPP: Dimethylallyl diphosphate
IPP: Isopenthyl diphosphate
GGPP: Geranyl geranyl pyrophosphate
DXS: 1-deoxy-D-xtylulose-5-phosphate
DXR: Deoxy-D-xtylulose-5-phosphate reductoisomerase
HDS: 4-hydroxy-3-methylbut-2-en-1-yl diphosphate synthase
HDR: 4-hydroxy-3-methylbut-2-en-1-yl diphosphate synthase
HDR: 4-hydroxy-3-methylbut-2-en-1-yl diphosphate reductase
GGPS: Geranyl geranyl pyrophosphate synthase
PSY: Phytoene synthase
PDS: Phytoene desaturase
Z-ISO: 15-cis-zeta carotene isomerase
ZDS: ζ-carotene desaturase
CRTISO: Carotenoid isomerase
LCYE: Lycopene epsilon cyclase
LCYB: Lycopene beta cyclase
CRTRB1: β-carotene hydroxylase
CCD1: Carotenoid cleavage dioxygenase 1
ZEP: Zeaxanthin epoxidase
FPKM: Fragments per kilo base per million reads
