# Supplementary material for: Genome-wide association analysis reveals new targets for carotenoid biofortification in maize
Source: Theor Appl Genet. 2015 Feb 18;128(5):851–64. doi: 10.1007/s00122-015-2475-3 (PMC4544543; doi:10.1007/s00122-015-2475-3)
Supplement: Supplementary file 2 — Supplementary material 2 (DOCX 243 kb) [file 122_2015_2475_MOESM2_ESM.docx]

FigureS1. Carotenoid biosynthetic pathway. Based on Babu et al (2013); Kandianis et al (2013), Gonzalez Jorge et al. (2013) and Arango et al (2014). Carotenoids are derived from products of glycolisis (blue box) and isoprenoid pathway (green blox). Provitamin A carotenoids are highlighted in orange.

CCD1 in red circles indicates the carotenoid substrates of CCD1 (Sun et al., 2008) and the corresponding catabolism products are highlighed in red. CCD4 is included as per the recent findings by Gonzalez-Jorge et al (2013).

Abbreviated intermediates: MEP methyl-erythriol 4-phosphate, DMAPP dimethylallyl diphosphate, IPP isopenthyl diphosphate, GGPP geranyl geranyl pyrophosphate. Enzymes are in black boxes and are defined as: DXS 1-deoxy-D-xtylulose-5-phosphate, DXR -deoxy-D-xtylulose-5-phosphate reductoisomerase, HDS 4-hydroxy-3-methylbut-2-en-1-yl diphosphate synthase, HDR 4-hydroxy-3-methylbut-2-en-1-yl diphosphate synthase, HDR 4-hydroxy-3-methilbut-2-en-1-yl diphosphate reductase, GGPS geranyl geranyl pyrophosphate synthase, PSY phytoene synthase, PDS phytoene desaturase, Z-ISO 15-*cis*-zeta carotene isomerase, ZDS ζ-carotene desaturase, CRTISO carotenoid isomerase, LCYE lycopene epsilon cyclase, LCYB lycopene beta cyclase, CRTRB1 β-carotene hydroxylase, CCD1 carotenoid cleavage dioxygenase 1, ZEP zeaxanthin epoxidase.

MEP

DXS, DXR

Glycolisis

HDS, HDR

DMAPP

GGPS

GGPP

PSY

Phytoene

PDS, Z-ISO, ZDS, CRTISO

Lycopene *Pseudo-ionone*

LCYE, LCYB

LCYB

α-carotene β-carotene *Pseudo- ionone*

CYP97A, ZmcrtRB3

*β-ionone*

CRTRB1,

ZmcrtRB3

Zeinoxanthin β-cryptoxanthin

CYP97A, HYDB

CYP97C

Lutein Zeaxanthin *Pseudo ionone, β-ionone,*

*3-hydroxy-β-ionone*

ZEP

Antheraxanthin

ZEP

Violaxanthin

ABA

Table S2. Known candidate genes of the carotenoid pathway in maize

| Chr | Position | Gene name | Abbreviation |
| --- | --- | --- | --- |
|  |  |  |  |
| 1 | 17,660,941-17,667,054 | Phytoene desaturase | *PDS1* |
|  |  |  |  |
| 2 | 15,865,938-15,868,219 | β-carotene hydroxylase | *HYD1* |
| 2 | 44,440,299-44,449,237 | Zeaxanthin epoxidase 1 | *ZEP1* |
| 2 | 207,236,994-207,238,335 | Geranylgeranyl pyrophosphate synthase 1 | *GGPS1* |
|  |  |  |  |
| 6 | 55,671,246-55,674,458 | Phytoene synthase | *PSY* |
| 6 | 146,378,412-146,382,661 | Deoxy xylulose synthase 1 | *DXS1* |
|  |  |  |  |
| 7 | 17,470,585-17,479,020 | ζ-carotene desaturase | *ZDS1* |
| 7 | 160,531,537-160,533,586 | Geranylgeranyl pyrophosphate synthase 2 | *GGPS2* |
|  |  |  |  |
| 8 | 138,882,594-138,889,812 | lycopene epsilon-cyclase | *LCYE* |
| 8 | 168,273,042-168,276,092 | Phytoene synthase 2 | *PSY2* |
|  |  |  |  |
| 9 | 152,086,899-152,092,882 | Carotenoid cleavage dioxygenase 1, syn. White cap 1 | *CCD1*, syn. *WC1* |
| 9 | 153,692,212-153,694,576 | β-carotene hydroxylase | *HYD5* |
|  |  |  |  |
| 10 | 4,705,086-4,705,639 | Phytoene synthase 3 | *PSY3* |
| 10 | 120,782,243-120,784,775 | Zeaxanthin epoxidase 2 | *ZEP2* |
| 10 | 136,057,214-136,060,219 | β-carotene hydroxylase | *CRTRB1* |

Adapted mainly from www.maizegdb.org. Chr: chromosome.

Table S3. Pearson phenotypic correlation coefficients among carotenoids (from means across three environments)

| Trait | LUT | ZEA | βCX | βC |
| --- | --- | --- | --- | --- |
| ZEA | 0.38** |  |  |  |
| βCX | 0.11 | 0.65** |  |  |
| βC | -0.11 | 0.22** | 0.38** |  |
| proVA | -0.09 | 0.38** | 0.65** | 0.94** |

LUT: lutein, ZEA: zeaxanthin, βCX: β-cryptoxanthin, βC: β-carotene, proVA: total provitamin-A.

All phenotypic values (y) were transformed to log_10_(y+1) scale prior to analysis. N=268.

** Significant at 0.01 level.

Table S4. Pearson phenotypic correlation coefficients among environments

| Trait | Environments | | |
| --- | --- | --- | --- |
|  | TL10-TL11 | TL10-AF12 | TL11-AF12 |
| LUT | 0.77** | 0.11ns | 0.20** |
| ZEA | 0.82** | 0.80** | 0.87** |
| βCX | 0.82** | 0.79** | 0.84** |
| βC | 0.84** | 0.76** | 0.84** |
| proVA | 0.84** | 0.78** | 0.84** |

LUT: lutein, ZEA: zeaxanthin, βCX: β-cryptoxanthin, βC: β-carotene, proVA: total provitamin-A.

TL10: Tlaltizapan, 2010 (HPLC method), TL11: Tlaltizapan, 2011 (HPLC), AF12: Agua Fria, 2012 (UPLC).

All phenotypic values (y) were transformed to log_10_(y+1) scale prior to analysis. N=268.

** Significant at 0.01 level, ns: not significant

Table S5. Potential donor lines for *DXS1*’s favorable allele associated with βCX content

| Line No. | Pedigree | βCX  (µg g^-1^) | proVA  (µg g^-1^) |
| --- | --- | --- | --- |
| CIM-SYN-7 | CML282 | 9.56 | 7.43 |
| CIM-SYN-71 | (Ac8730SR-##-124-1-5-B-1-#/[BETASYN]BC1-16-2-3-1-1)-B | 2.25 | 3.76 |
| CIM-SYN-89 | (KUI2007-B-B-B-B)-B | 7.61 | 8.29 |
| CIM-SYN-121 | CML168 | 7.77 | 8.78 |
| CIM-SYN-161 | ([[EV7992]C1F2-430-3-3-3-X-7-B-B/CML202]-6-2-2-3-B*3/[BETASYN]BC1-10-1-1-1-1-B-B)-B | 9.10 | 8.00 |
| CIM-SYN-354-2 | (P591c41y2GENF12-1-1-1-B-B-B-B//P591c41y2GENF12-1-1-1-B-B-B/KUIcarotenoidsyn-FS25-3-2-B)-B-24-3 | 6.45 | 7.90 |
| CIM-SYN-356-4 | (P591c41y2GENF12-1-1-1-B-B-B-B//P591c41y2GENF12-1-1-1-B-B-B/CML297)-B-7-2 | 6.61 | 8.37 |
| CIM-SYN-418-66 | (KUIcarotenoidsyn-FS17-3-1-B-B/CML356//CML305)-2-1 | 7.13 | 5.39 |

Table S6. Potential donor line for *CCD1* ‘s favorable allele associated with βCX content

| Line No. | Pedigree | βCX  (µg g^-1^) | proVA  (µg g^-1^) |
| --- | --- | --- | --- |
| CIM-SYN-35 | (((CML150xCML451)-B-33-3-1-B-Bx(CML176xCL-G2501)-B-43-1)-B-38-1-2-1-1-B)-B | 8.17 | 10.53 |
| CIM-SYN-76 | CML32 | 5.89 | 6.88 |
| CIM-SYN-107 | ((KU1409/SC55/KU1409)-S2-12-1-B-B)-B | 4.06 | 4.40 |
| CIM-SYN-154 | CML285 | 5.90 | 7.06 |
| CIM-SYN-168 | (LAMA2002-46-2-B)-B | 5.68 | 6.62 |
| CIM-SYN-177 | (B97-B)-B | 5.05 | 5.28 |
| CIM-SYN-400-48 | (CML300/CML486)-7-2-2-B | 6.00 | 6.52 |
| CIM-SYN-401-49 | ((DTPYC9-F65-2-3-1-1-B-BxDTPYC9-F65-2-2-1-1-B-B)xDTPYC9-F86-1-1-1-1-B-B-B)-B-B-7-1-B | 6.25 | 8.52 |
| CIM-SYN-402-50 | (CML305/CML486)-8-1-1-B | 6.36 | 6.62 |
| CIM-SYN-425-73 | (CarotenoidSyn3-FS5-1-5-B-B/CML353//CML486)-6-1 | 8.53 | 8.30 |
| CIM-SYN-322 | NC486/(NC486/CarotenoidSyn3-FS5-1-5-B-B//KUIcarotenoidsyn-FS17-3-2-B-B-B)-2-3 | 4.68 | 4.22 |

Table S7. RNA expression (Sekhon et al. 2013) of the genes located inside or nearby significant SNPs

| No. | Trait | Marker  (Chromosome_  Position) | Nearby Gene | Annotation | Gene Position Left | Gene Position Right | 16DAP  Endosperm  Expression (FPKM) |
| --- | --- | --- | --- | --- | --- | --- | --- |
| 1 | BC | S1_280088079 | GRMZM2G156800 | MAPKKK5 | 280,087,792 | 280,097,855 | 52.5 |
| 2 | BC | S2_2680579 | GRMZM2G010555 | Alternative oxidase | 2,675,436 | 2,685,261 | 12.8 |
| 3 | BC | S2_212648728 | GRMZM2G064640 | 40S ribosomal protein S9 | 212,644,796 | 212,647,696 | 2104.3 |
| 4 | BC | S10_133820657 | GRMZM2G026391 | metal-nicotianamine transporter YSL16 | 133,816,855 | 133,820,703 | 16.8 |
| 5 | BC | S10_135911532 | GRMZM2G162640 | Protein binding protein | 135,910,598 | 135,912,870 | 27.0 |
| 6 | BC | S10_135911532 | GRMZM2G152135 | Beta-carotene hydroxylase 1* | 136,057,099 | 136,060,219 | 1148.5 |
| 7 | BCX | S2_185172752 | GRMZM2G162177 | Hydroxyproline-rich glycoprotein | 185,170,411 | 185,177,602 | 35.3 |
| 8 | BCX | S2_207044142 | AC194970.5_FG001 | geranylgeranyl pyrophosphate synthase 1* | 207,236,994 | 207,238,335 | 81.3 |
| 9 | BCX | S2_224340705 | GRMZM2G169095 | Xaa-Pro aminopeptidase 1 | 224,332,393 | 224,344,331 | 447.5 |
| 10 | BCX | S9_151998412 | GRMZM2G126839 | RNA recognition motif family protein | 151,992,697 | 151,999,977 | 35.5 |
| 11 | BCX | S9_151998412 | GRMZM2G057243 | 9,10-9,10 carotenoid cleavage dioxygenase 1* | 152,086,898 | 152,092,882 | 13.2 |
| 12 | BCX | S10_136115355 | GRMZM2G016477 | LRR receptor-like serine/threonine-protein kinase | 136,114,578 | 136,118,747 | 19.1 |
| 13 | LZ | S2_6340709 | GRMZM2G054225 | DNA-directed RNA polymerase | 6,335,271 | 6,346,708 | 34.7 |
| 14 | LZ | S2_56927499 | GRMZM2G388539 | OJ000126_13.5 protein | 56,923,760 | 56,929,922 | 52.4 |
| 15 | LZ | S7_143524503 | GRMZM5G852968 | Triosephosphate isomerase | 143,524,117 | 143,527,994 | 3.1 |
| 16 | LZ | S8_138523563 | GRMZM2G106479 | Integral membrane protein | 138,519,519 | 138,524,207 | 671.9 |
| 17 | LZ | S8_172409688 | GRMZM2G173868 | ER degradation-enhancing alpha-mannosidase-like 1 | 172,403,830 | 172,424,265 | 71.5 |
| 18 | LZ | S9_130410559 | GRMZM2G078238 | MAP kinase activating protein | 130,405,594 | 130,414,641 | 53.4 |
| 19 | LZ | S10_119414483 | GRMZM2G446201 | ARGOS | 119,412,829 | 119,414,563 | 92.6 |
| 20 | ZEA | S2_57643868 | GRMZM2G074238 | Catalytic/ hydrolase | 57,643,339 | 57,646,559 | 4.0 |
| 21 | ZEA | S6_154891169 | GRMZM2G145662 | Conserved gene of unknown function | 154,887,524 | 154,891,678 | 46.6 |
| 22 | ZEA | S7_160069429 | GRMZM2G102550 | geranylgeranyl pyrophosphate synthase 2* | 160,531,536 | 160,533,586 | 11.1 |
| 23 | ZEA | S9_133887810 | GRMZM5G862107 | 30S ribosomal protein S1 | 133,886,704 | 133,891,175 | 166.7 |
| 24 | ZEA | S10_90488005 | GRMZM2G166694 | CUL1 | 90,481,741 | 90,492,880 | 370.3 |

DAP: days after pollination. FPKM: fragments per kilo base per million reads.

* Genes involved in the carotenoid pathway.

Figure S2. Distributions of phenotypic values of each trait in original scale

| **55K** | **GBS** |
| --- | --- |
| 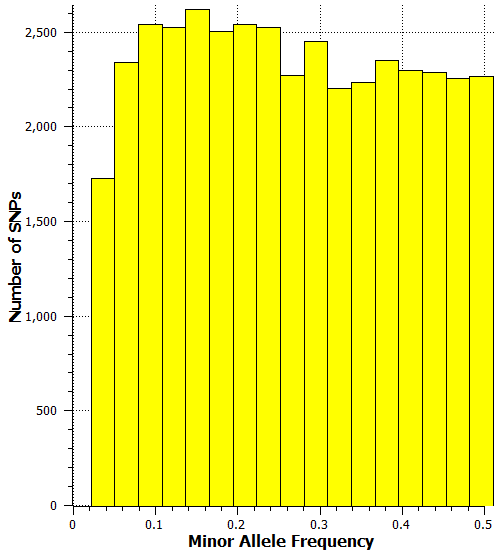 | 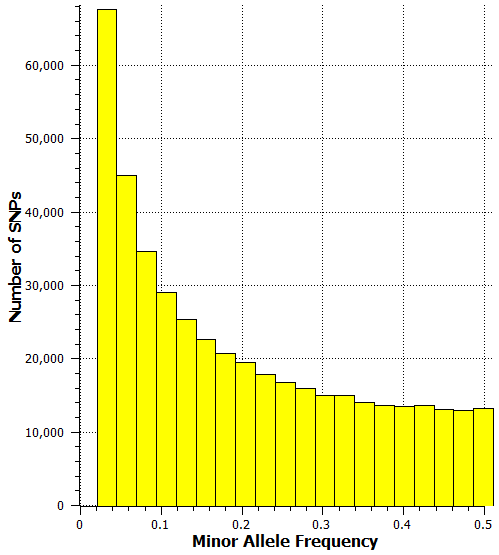 |
|  |  |
| **55K+GBS** |  |
| 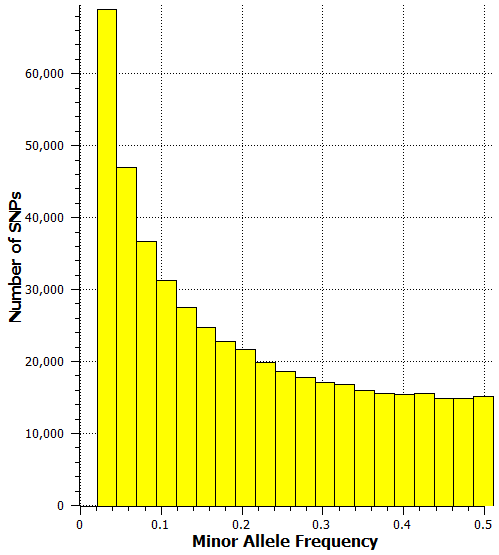 |  |

Figure S3. Distribution of minor allele frequency of the 55K, GBS, and 55K+GBS data


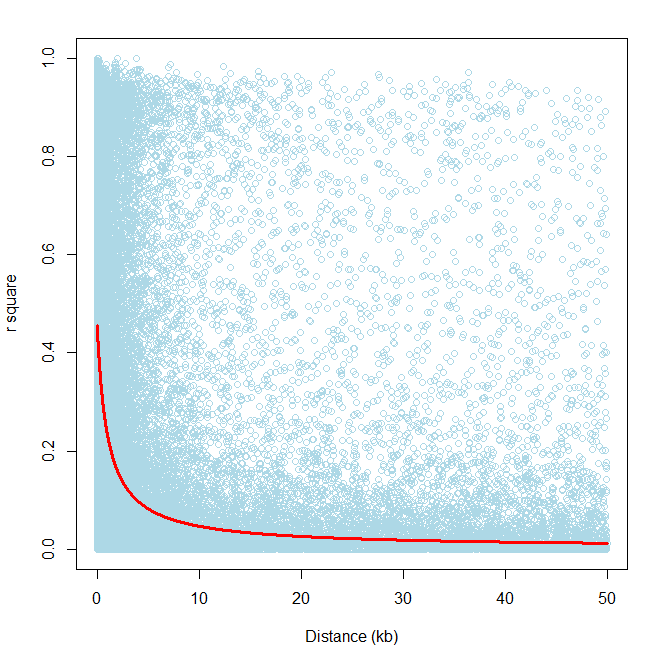


Figure S4. LD plot showing rate of genome-wide LD decay based on adjacent pair-wise genetic and physical distances calculated using high quality subset of GBS data (pairs with distance > 50 kb are not shown)

Figure S5. Average adjacent pair distances among SNPs in which LD decayed at r^2^ = 0.2 and 0.1; chromosome-wise and genome-wide (GW)

| **G Model** | **G+Q (FELM) Model** |
| --- | --- |
| 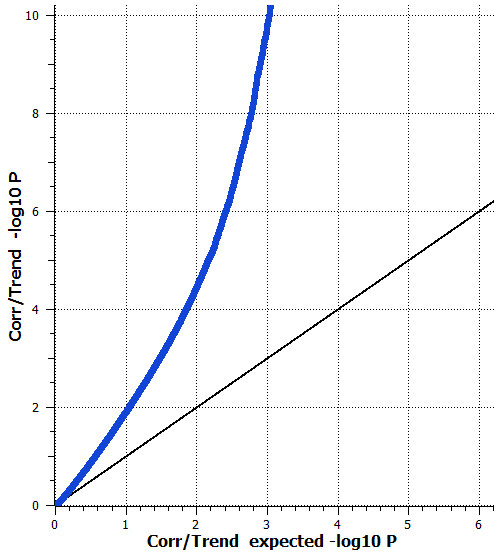 | 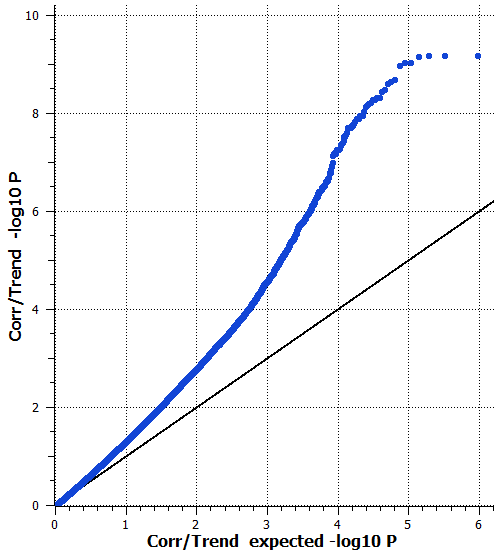 |
|  |  |
| **G+K Model** | **G+Q+K (MLM) Model** |
| 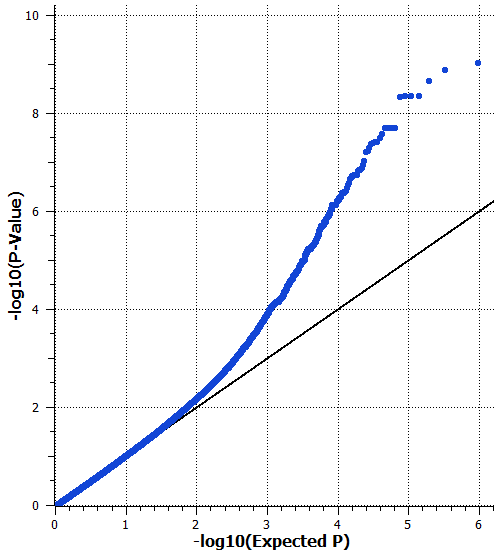 | 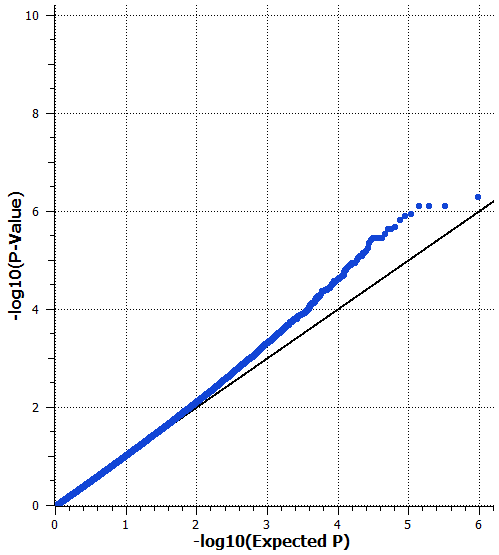 |

Figure S6. Q-Q plots of observed versus expected -log_10_(P-values) plots for β-carotene, evaluating four association mapping models in the 55K+GBS combined data. G = genotype (fixed), Q = ten principal components (fixed), K = kinship matrix (random). The phenotypic values (y) were transformed to log_10_(y+1) prior to analyses
